# Computer-Aided Detection and Diagnosis of Neurological Disorder

**DOI:** 10.7759/cureus.28032

**Published:** 2022-08-15

**Authors:** Shreyash Huse, Sourya Acharya, Samarth Shukla, Harshita J, Ankita Sachdev

**Affiliations:** 1 Medicine, Jawaharlal Nehru Medical College, Datta Meghe Institute of Medical Sciences (Deemed to be University), Wardha, IND; 2 Pathology, Jawaharlal Nehru Medical College, Datta Meghe Institute of Medical Sciences (Deemed to be University), Wardha, IND; 3 Surgery, Jawaharlal Nehru Medical College, Datta Meghe Institute of Medical Sciences (Deemed to be University), Wardha, IND

**Keywords:** comprehensive learning, nervous system illnesses, diagnosis advancements, neurological problems, computer-aided diagnosis

## Abstract

Nowadays, neurological problems are more regular, representing a worry to pregnant ladies, guardians, healthy babies, and kids. Neurological problems emerge in a wide assortment of structures, each with its arrangement of beginnings, inconveniences, and results. The conclusion of neurological illnesses is an evolving concern and predominantly troublesome difficulty for current medication. Current diagnosis advancements (e.g., MRI and EEG) produce immense information (in proportion and aspect) as location, checking, and therapy of nervous system illnesses. As a common rule, investigation of that enormous clinical information is performed physically by specialists to distinguish and figure out the irregularities. It is a genuinely troublesome errand for an individual to collect, make due, investigate, and absorb enormous amounts of information through visible review. As an outcome, the specialist has been requesting electronic conclusion frameworks known as “computer-aided diagnosis” that can consequently identify the nervous system irregularities utilizing the essential clinical information. This framework further develops uniformity of findings, builds treatment outcomes, protects lives, and lessens price and time. As of late, few examinations have improved the computer-aided design frameworks for the executives of enormous clinical information for determination appraisal. This paper investigates the difficulties of tremendous clinical information giving. This article fundamentally evaluated and looked at the exhibition of existing AI and comprehensive learning approaches for identifying nervous system illnesses. A far-reaching piece of this concentration also shows different modalities and illness-determined datasets that identify and record pictures, signs, addresses, and so forth. Restricted related works are additionally summed up on nervous system illnesses, as this space has essentially less work zeroed in on illness and recognition rules. A portion of the standard assessment measurements is likewise introduced in this review for improved outcome examination.

## Introduction and background

Medical services have been developed into a significant share of the human way of life. Afterward, the change and improvement of medical services frameworks have become exceptionally prevailing concerning advancements. Recognizing illnesses has likewise become highly reliant upon biomedical advances, for example, ultrasound, x-beams, molecule bars, and x-rays [1]. It is assessed that 34.5 million individuals have dementia, with 7.69 million new cases consistently; Alzheimer's disease (AD) is the most extensively recognized cause of dementia, which may add to 65%-75% of cases [2].

**Figure 1:**
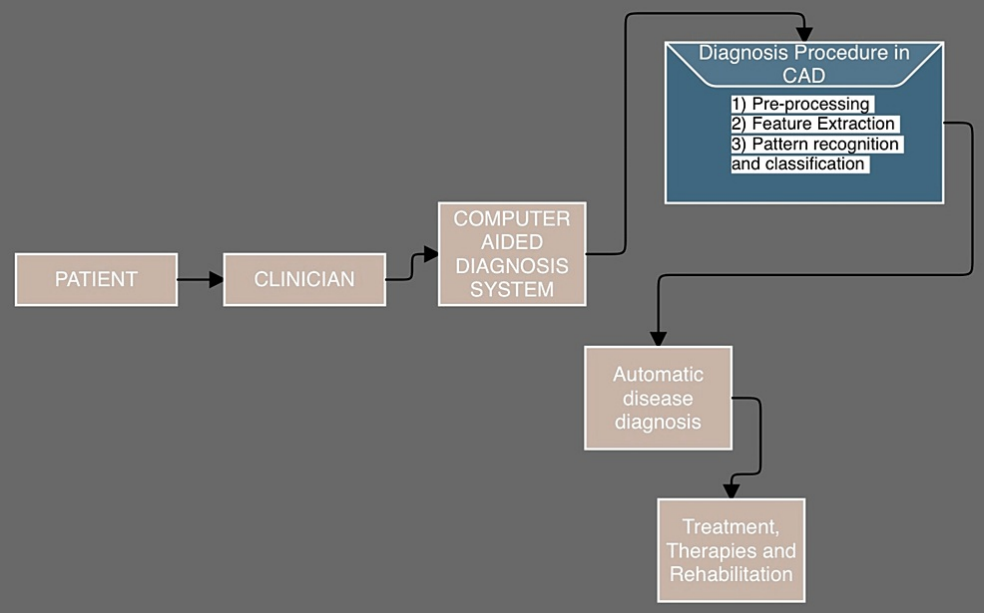
Computer-aided diagnosis system for robotized detection of nervous system abnormalities.

Neurological illnesses are a section of human issues that distinguish inconveniences of the mind. Neurological ailments, called mind, social, or mental problems, influence individual capacities to walk, talk, learn, and move [3]. As the mind is the control focus of the human nerves, influencing the mind can compromise one's life. Mindfulness of these illnesses has decreased the death rate; notwithstanding, a few ongoing neurological illnesses can cause super durable and halfway inability or languishing. The worldwide predominance of these problems represented 10.3% of the cases. Moreover, these diseases have a more causality pace of 17.9 % each year. These rates demonstrate that nervous system and neurodevelopmental problems have more handicap rates than other human issues [4]. Clinical picture information ranges from a couple of megabytes for a solitary report to too many megabytes per case (for example, thin slice C.T. contains up to 2,600+ sweeps per case). Such information requires enormous capacity limits whenever put away for a long haul. Because of the volume, speed, and intricacy of the clinical data, it is tough for the specialists to aggregate, make due, break down and absorb the enormous importance of information for finding, treatment evaluation, and arranging. Incorporating a high amount of physiological data is an excellent test for specialists to convey clinical suggestions. Supporting clinical specialists or nervous system specialists during the time spent finding the correct determination to speculation as quickly as possible is attractive to work on a patient's result. As a general rule, the investigation of those enormous data measures is performed physically through visual examination by nervous system specialists/specialists to recognize and comprehend anomalies from clinical imaging and signal information. The procedures utilized by computer-aided design systems incorporate information handling, highlight extraction, and characterization as shown in Figure 1. The computer-aided design arrangements help experts assess enormous clinical information, further developing conclusion precision and consistency while lessening examination time. The computer-aided design framework is low-cost and productive, and experts could use it to determine and treat neurological diseases as an emotionally supportive network. The procured clinical information (e.g., clinical picture or clinical sign information) was handled during the pre-handling time frame to eliminate clamor and lessen the intricacy and calculation season of computer-aided design calculations. One of the fundamental components of the computer-aided design framework is the element extraction area, which extricates infection biological indicators from the source information. The removed element aims to contribute to the sectionalization model for dispensing the possibility of most accessible classifications (e.g., solid or typical) because of a sectionalization in the characterization procedure for computer-aided design frameworks [5]. As of late, a high-level thought on robotized computer-aided diagnosis (CAD) framework presented by the specialists/nervous system specialists for identifying the neurological anomalies from the enormous clinical information. The calculations of powerful computer-aided design frameworks are created using procedures and hypotheses of the example acknowledgment field. Accordingly, computer-aided design is involved as one of the examples of acknowledgment fields [6].

## Review

Current clinical advances for clinical information assortments and difficulties in clinical huge information examination

As of now, neurological illnesses are analyzed by utilizing different clinical methods, for example, electroencephalography (EEG), computerized tomography (CT sweep or Feline output, MRI, EMG), positron emission tomography (PET sweep or PET symbolism), arteriogram (additionally known as an angiogram) and single photon emission computed tomography (SPECT). These indicative tests assist doctors in affirming and preclude the existence of any nervous system problem and other ailments. To analyze cerebrum-related illnesses, for example, epilepsy, specific seizure issues, degenerative problems, sleep issues, mental imbalance, brain tumors, and headaches, EEG is utilized to record synapse action through the skull for concentrating on the practical conditions of the mind to help doctors for distinguishing and checking cerebrum irregularities [7]. Varieties or anomalies in mind waves suggest various kinds of neurological issues. To recognize mind anomalies, a CT or feline output is utilized to see the transaction pictures of the body utilizing an x-ray and PC [8].

MRI results assist doctors with diagnosing torn tendons, growths, course (bloodstream) issues, eye infection, irritation (e.g., joint inflammation), and contamination. MRI checks are additionally used to recognize; screening for degenerative issues like multiple sclerosis can also archive the cerebrum injury. Suppose the doctor needs to explore the mind in real life (e.g., talking or operating activities) and spot the spaces of the mind that becomes dynamic and note how long they stay dynamic [9]. SPECT analysis is likewise requested as a chase up to an x-ray to analyze cancers, contaminations, deteriorating spinal infection, and stress breaks. To recognize unusual electrical action of muscle that can happen in a large number of illnesses and conditions, for example, amyotrophic lateral sclerosis, carpal tunnel condition, muscular dystrophy, sciatic nerve dysfunction, neuropathy, aggravation of muscles, and an EMG is utilized to record the electrical action of muscles [10]. To distinguish unique heart issues, such as respiratory failure, coronary heart illnesses, and stroke, ECG tracks the heart's electrical movement to comprehend how the heart functions [11]. These clinical innovations produce gigantic amounts of detailed and high-aspect information that are a significant hotspot for diagnosing neurological illnesses, therapy, and treatment arranging. The superb clinical information examination has the potential to be a powerful instrument, yet execution can present a test. It requires careful information examination which can support vide bona fide, precise, and reliable data for good dynamic in sickness conclusion. Practically speaking, a large portion of the case translations of that information is achieved visually by specialists/neuro specialists [12].

CAD framework for computed detection of neurological disorders

As of late, computer-aided design is becoming exceptionally well known in clinical and analytic imaging for programmed distinguishing anomalies from enormous clinical information sources. The College of Chicago presented the essential idea of computer-aided design during the 1980s; the thought was to give a computed output as a “2nd assessment” to help specialists in deciphering clinical information; thus, the precision and uniformity of determination as it may be enhanced, and the examination time can be decreased [13,14]. 

Epilepsy and epileptic seizure detection 

Epilepsy is the most well-known and disturbing nervous system disorder worldwide. Epilepsy is portrayed as intermittent seizures [15]. Seizures are characterized as an unexpected swap in the electronic working of the cerebrum, coming about in adjusted ways of behaving, for example, passing out, jerky developments, transitory loss of breath, and cognitive decline. The EEG is a significant clinical device that contains essential data for understanding epilepsy [16]. Its main indication is the epileptic attack, which could incorporate a separate piece of the brain partially or the total cerebral mass generally. Throughout recent years, various epileptic seizure identification and expectation calculations have been created in a few nations worldwide. All the more, as of late, Shen et al. presented a strategy based on an outpouring of wavelet-rough entropy for highlight extraction in the epileptic EEG signal order [17]. They tried three existing techniques for characterization: support vector machine (SVM), k-nearest neighbor (kNN), and radial basis function neural network (RBFNN), to figure out which has the best presentation in like flowed EEG examination framework. Acharjee and Shahnaj utilized 12 Cohen class works to change EEG information to work with the time recurrence investigation. The changed information figured out a component vector comprising extraordinary power and particular degeneration, and the component vector was taken care of by an artificial neural network (ANN) sectionalization [18]. Silly et al. presented a mechanized methodology in light of straightforward irregular testing procedures and most miniature square help vector machines (LS-SVM) to order epileptic EEG signals [19]. In one more work, Siuly and Li fostered another calculation for highlighting extortion taking into consideration the fluctuation of the perceptions inside a period window known as the ideal portion approach. Then, at that point, the extorted highlights were surveyed by different multiclass least square help vector machines, ordering epileptic EEG signals [20]. Aslan and the team executed a review to look at epileptic cases by creating a characterization technique. The order interaction was fractional and essentially summed up epilepsy using R.B.F.N.N. and multilayer perceptron neural network (MLPNNs) [21].

Multiple sclerosis detection 

Multiple sclerosis (MS) is a chronic condition of the central nervous system, which comprises the cerebrum along with the spinal cord. An abnormal state can be tolerably associated with debilitating. There are numerous potential reasons for MS, including infections, immune system issues, natural factors, and hereditary variables. MS influences an expected 2.3 million individuals around the world. Ladies are impacted over two times as frequently as men, per the Public MS Society. Various mechanized techniques are proposed for identifying MS sores because of a few layered (three-dimensional) MRI pictures. Alfano et al. fostered a mechanized approach in light of relaxometry and mathematical highlights for the order of MS sores from three-dimensional MRI pictures [22]. Boudreau et al. utilized the FCM calculation to 1.5 T two-layered (2-D) MRI pictures for ordering ordinary and unusual cerebrum structures [23]. Leemput et al. planned a robotized strategy using a power-based tissue characterization and a stochastic model to recognize MS sores from three-dimensional pictures [24]. Zijdenbos et al. fostered a computer-aided design structure for the pipeline examination of MS sores in MRI information [25]. Khayati et al. proposed a robotized technique for the division of MS sores in mind, utilizing a versatile blend strategy and a Markow irregular area model in three-dimensional MRI pictures. Their proposed technique depended on a Bayesian sectionalization to get and update the category contingent likelihood thickness capability and an earlier likelihood of each class [26].

Dementia, Parkinson’s diseases, AD diagnosis

Dementia alludes to a gathering of neurodegenerative issues brought about by the steady neuronal brokenness and the demise of synapses. This problem could be characterized medically as a condition that gives rise to a decrease in mental space (i.e., attentiveness, remembrance, leadership capability, visual-spatial capacity, and speech) expected in the older [27]. Under the American Institute of Nervous system science rundown report, 10% of individuals over 56 and half of those over 76 face dementia [28]. In 2012, it was assessed that almost 250,000 individuals in Australia region go through dementia out of an all-out people count of 22.5 m. The present figure is expected to increase to 850,000 by 2050 [29]. Dementia is grouped into AD, Parkinson's illness, dementia with Lewy bodies, Creutzfeldt-Jakob infection, typical tension hydrocephalus, vascular dementia, and fore transient dementia [30]. AD is the main and regular kind of dementia. Of every referenced kind of dementia, two-third of the demented cases experience the ill effects of AD. In this segment, we briefly survey computer-aided design techniques for identifying dementia, AD and Parkinson’s diseases from medical picture data and signal data. There are countless programmed PC help strategies produced for recognizable proof of dementia. Koikkalainen et al. finished a broad review on various created strategies for computer-aided design framework for identifying dementias utilizing preliminary MRI information [31]. A broad set of Hirata et al. created programming because of the voxel-based detailed district investigation for AD, which can naturally dissect 3D MRI information as a progression of division, physical normalization, and regular utilizing a product and Z-score investigation [32]. Li and his team utilized an SVM to characterize the hippocampal volume changes in the AD and separation of AD patients from sound control subjects [33]. Kloppel et al. fostered a Computer-aided design strategy for determining AD from x-ray examines from two unique habitats and two distinct gears utilizing a direct help vector. In their strategy, x-ray pictures were sectioned into grey matter (GM), white matter, and CSF utilizing SPM [34]. Colliot et al. fostered a computerized segmentation strategy to help recognize patients with AD, mild cognitive impairment (MCI), and older controls [35].

Various investigations have been directed on the computer-aided design framework to manage EEG changes related to dementia. The specialists created computer-aided design techniques to distinguish the level of seriousness of dementia, and a few investigations support the possibility for EEG to distinguish dementia in its beginning phases. Henderson et al. distinguished early dementia presence in EEGs with high awareness and particularity for the model [36] EEG might assume a significant part in recognizing and ordering dementia in light of its critical effect based on dementia anomalies in conditions of the beat movement.

Sleep disorders diagnosis

The expression “sleep illnesses” alludes to a scope of states which bring about anomalies during rest. The most recognized problem is rest apnea. Apnea implies a temporary pause in breathing. Rest apnea happens when the boundaries of the windpipe meet up throughout rest, closing off the higher aviation route. Respiration ceases for a while until the mind enlists the absence of respiration or a lower oxygen level and afterward makes a little reminder [37]. The complete term for this situation is obstructive sleep apnea (OSA). Focal rest apnea is another exciting type of breathing aggravation during rest. It is made by disturbing the components which check the rate along with profundity of relaxation. Corpulence is quite possibly the most common reason for rest apnea. Various calculations have been proposed to handle the issue of programmed OSA identification. Azarbarzin and Mousavi fostered a computer-aided design strategy because of linear discrimination analysis, where zero-crossing rate and pinnacle recurrence were determined from wheezing sound signs [38]. Schlotthauer et al. utilized the exact decay of heartbeat oximetry signs for robotized OSA. screening [39]. Varon et al. proposed head parts of the QRS complex as highlights for recognizing OSA [40]. Chen et al. utilized a serious record of OS with help vector machines for computer helped rest apnea identification. Single lead E.C.G. signals were utilized in the present study [41]. In another work, Chen and team carried out a piece thickness assessor on highlights separated from sectioned R.R. spans to perform characterization of OSA [42].

## Conclusions

The dramatic impact of nervous system sickness pathologies on living status is a developing worry. Current advances for detecting nervous system illnesses produce extraordinary measures of clinical information like clinical pictures or electrical signs information. Deciphering those pictures or signals is a definitive “large data” issue. Clinical picture investigation, signal handling, what is more, and the combination of physiological information face comparative difficulties in managing different enormous information sources. This paper considers how an astute calculation framework can oversee crucial clinical information to conclude neurological illnesses. An overview of ongoing detailed computer-aided design strategies was to be given in this paper. Neurologists anticipate that computer-aided design frameworks can help diagnose neurological illnesses by giving valuable data. Although there have been broad examinations into the improvement of different computer-aided design frameworks for programmed screening sicknesses, the specialists are still unfit to involve every one of them in their emotional cycle because of the absence of effortlessly utilized internet-based strategies. Besides, in some cases, wrong decisions regarding element extraction strategies and order techniques in the computer-aided design frameworks can make pointless outcomes to figure out illness. To make powerful computer-aided design frameworks, an improvement of AI calculations is likewise crucial for excellent order work also analysis. In writing, it has been seen that the created computer-aided design frameworks are engaged separately in most cases. At the same time, the specialists require online computer-aided design frameworks for constant assessment. Besides, the best strategies endure from a compromise between exactness and productivity. Subsequently, the upgrades in the computer-aided design logical frameworks are required to examine the enormous amount of information in a clinical setting. Accordingly, all together, to create significantly more exact differential analytic frameworks, further examination is expected in the accompanying bearings: (1) creating effective internet based computer aided design frameworks; (2) growing more broad element extraction strategies; (3) creating rigorous characterization techniques; and (4) all in all appropriate size of preparing alongside testing informational collection as enormous preparation information increment order exactness. Through tending to these concerns, a precise computer-aided design model could be produced to analyze nervous system anomalies that would help the specialists with the ideal determination.
